# Real-world implementation of video-observed therapy in an urban TB program in the United States

**DOI:** 10.5588/ijtld.21.0170

**Published:** 2021-08-01

**Authors:** A. Perry, A. Chitnis, A. Chin, C. Hoffmann, L. Chang, M. Robinson, G. Maltas, E. Munk, M. Shah

**Affiliations:** 1 Johns Hopkins Bloomberg School of Public Health, Baltimore, MD; 2 Tuberculosis Control Section, Alameda County Public Health Department, San Leandro, CA; 3 Division of Infectious Diseases, Johns Hopkins School of Medicine, Baltimore, MD, USA

**Keywords:** medication adherence, telemedicine, tuberculosis, vDOT

## Abstract

**BACKGROUND:**

Video directly observed therapy (vDOT) was introduced to increase flexibility and meet patient-specific needs for TB treatment. This study aimed to assess the reach and effectiveness of vDOT for TB treatment under routine conditions in Alameda County, CA, USA, a busy, urban setting, from 2018 to 2020.

**METHODS:**

We prospectively evaluated routinely collected data to estimate 1) reach (proportion of patients initiated on vDOT vs. in-person DOT); and 2) effectiveness (proportion of prescribed doses with verified administration by vDOT vs. in-person DOT).

**RESULTS:**

Among 163 TB patients, 94 (58%) utilized vDOT during treatment, of whom 54 (57%) received exclusively vDOT. Individuals receiving vDOT were on average younger than those receiving in-person therapy (46 vs. 61 years; *P* < 0.001). The median time to vDOT initiation was 2.2 weeks (IQR 1.1–10.0); patients were monitored for a median of 27.0 weeks (IQR 24.6–31.9). vDOT led to higher proportions of verified prescribed doses than in-person DOT (68% vs. 54%; *P* < 0.001). Unobserved self-administration occurred for all patients on weekends based on clinic instructions, but a larger proportion of doses were self-administered during periods of in-person DOT than of vDOT (45% vs. 24%; *P* < 0.001).

**CONCLUSION:**

A TB program successfully maintained vDOT, reaching the majority of patients and achieving greater medication verification than in-person DOT.

## Abstract (FR)

**CONTEXTE:**

Le traitement sous observation vidéo (vDOT) stratégie vDOT a été mise en place afin d’accroître la flexibilité et de satisfaire certains besoins spécifiques des patients dans le cadre du traitement de la TB. Cette étude (2018–2020) avait pour objectif d’évaluer la portée et l’efficacité de la stratégie vDOT dans le traitement de la TB, dans des conditions de routine, en milieu urbain dense dans le Comté d’Alameda (Californie, É tats-Unis).

**MÉTHODES:**

Nous avons évalué de manière prospective les données recueillies en routine pour estimer 1) la portée (pourcentage de patients mis sous vDOT vs. DOT en personne) ; et 2) l’efficacité (pourcentage de doses prescrites avec administration vDOT vs. DOT en personne).

**RÉSULTATS:**

Parmi 163 patients atteints de TB, 94 (58%) ont eu recours au vDOT pendant leur traitement, dont 54 (57%) de manière exclusive. Les sujets sous vDOT étaient en moyenne plus jeunes que ceux sous DOT en personne (46 vs. 61 ans ; *P* < 0,001). Le délai médian avant instauration du vDOT était de 2,2 semaines (IQR 1,1–10,0). Les patients ont été suivis pendant une durée médiane de 27,0 semaines (IQR 24,6–31,9). La stratégie vDOT a permis d’accroître le pourcentage de doses prescrites contrôlées par rapport à la stratégie DOT en personne (68% vs. 54% ; *P* < 0,001). Une auto-administration non-observée du traitement, avec instructions cliniques, avait été mise en place le weekend pour tous les patients, mais un pourcentage plus important de doses ont été auto-administrées pendant les périodes sous DOTen personne que pendant les périodes sous vDOT (45% vs. 24% ; *P* < 0,001).

**CONCLUSION:**

Ce programme de lutte contre la TB a réussi à appliquer la stratégie vDOT sur la durée, parvenant à inclure la majorité des patients et à atteindre des taux de contrôle de traitement plus élevés qu’avec la stratégie DOT en personne.

TB is the leading cause of infectious disease death globally and poses significant burden to individual and public health.^1^,^2^ While TB is treatable and curable, treatment requires taking several medications for extended durations.^1^,^3^,^4^ Directly observed therapy (DOT) has become the standard of care and codified into law in parts of the United States.^5^–^14^ Current Centers for Disease Control and Prevention/Infectious Diseases Society of America/American Thoracic Society (CDC/IDSA/ATS) treatment guidelines recommend utilizing DOT as part of a comprehensive, patient-centered approach to TB case management; however, DOT may present challenges in scheduling and resources.^15^–^19^ Thus, while TB guidelines recommend daily therapy for 7 days a week, many health departments dose or verify TB medications during business days or intermittently, given the typical logistical challenges.^19^,^20^

An emerging approach to provide patient-centered treatment verification is video DOT (vDOT).^7^,^19^,^21^ vDOT utilizes technological advancements to facilitate DOT for patients remotely through computer or mobile devices, in real-time (synchronous) or recorded (asynchronous).^19^,^22^ Previous trials evaluating the effectiveness of vDOT have been promising, reporting high adherence rates as compared to in-person DOT.^16^,^22^,^23^ vDOT has been adopted for routine use in the United States, with almost half of TB programs having used vDOT in some capacity in 2015, and 36% planning to implement a vDOT program in the following year.^24^

A previous prospective study within five health jurisdictions in California measured treatment adherence for patients using vDOT vs. traditional DOT under study conditions.^22^ This study observed a higher fraction of expected doses observed using vDOT than in-person DOT, along with high patient satisfaction and acceptability.^22^ However, it has not been established whether such findings would be maintained under programmatic conditions. It is also unknown whether staff uptake and recommendations for vDOT might change with growing experience and comfort with technology. We sought to assess the reach and effectiveness of vDOT compared to in-person DOT under routine implementation in a busy, urban TB program at the Alameda County Public Health Department (ACPHD) in California, USA.

## METHODS

### Overview

We conducted a pragmatic, prospective observational evaluation of TB treatment monitoring measured by self-report, in-person DOT, and asynchronous vDOT under routine conditions at the ACPHD TB program. Our objective was to assess the reach and effectiveness of programmatic vDOT implementation utilizing the emocha Health Insurance Portability and Accountability Act-compliant mobile app (emocha Mobile Health Inc, Baltimore, MD, USA) for TB treatment monitoring under routine programmatic conditions. Protocols were approved by the ethics committees at Johns Hopkins University, Baltimore, MD, USA, and California Department of Health, Sacremento, CA, USA.

### Study population

We abstracted routinely collected data from patient medical records for patients receiving treatment for active TB through the ACPHD TB Control Program, who were ≥ 18 years of age and with ≥2 months of therapy remaining. We excluded pediatric patients as considerations for DOT and vDOT may be different in that population warranting a dedicated study; individuals with less than 2 months remaining were excluded to ensure that included participants had sufficient follow-up time to measure adherence. The program introduced vDOT as a pilot program in 2017 and officially licensed emocha software for routine usage in late 2018. We subsequently evaluated data on all patients who were in care from September 1^st^, 2018 to August 31^st^, 2019, with follow-up available until February 28^th^, 2020. The determination of modality (i.e., self-report, vDOT, in-person DOT) was made exclusively by the health department and patient care team using locally developed protocols and in accordance with California Department of Health (CDPH) guidelines.^25^,^26^ The local practice, in accordance with CDPH guidance, was to consider vDOT for all patients in whom the program felt would benefit from observed therapy.^7^,^26^. Individualized decisions on DOT modality were made by the program and the patient using a shared decision-making paradigm inclusive of patient considerations and preferences, including patient willingness and ability to participate in vDOT procedures. An initial assessment on DOT modality (in-person/field-based vs. vDOT) is made by the public health nurse (PHN), and includes factors such as willingness and interest in using technology for monitoring, degree of communication between patient and health care team, and patient preferences based on schedules, distance to clinic, and other individualized factors. There were no restrictions on timing of vDOT initiation or prerequisite demonstration of adherence prior to vDOT initiation. There were no a priori formal exclusion criteria for vDOT usage on the basis of alcohol or substance use, preferred language, or homelessness, or any other patient sociodemographic features, or microbiological criteria. After the initial PHN assessment, a Senior Program Specialist makes a secondary assessment during enrollment into TB care to determine feasibility of vDOT and in-person DOT. Patients and providers are free to switch DOT modalities (in-person to vDOT, and vice versa) at any time based on individual circumstances.

### TB treatment

All patients received case-management and treatment decisions per routine protocols irrespective of DOT modality; this included case-management phone calls or visits following missed doses or reported side effects. Treatment decisions were clinician-directed according to CDPH and CDC guidelines.^15^,^25^,^26^ Drug regimens generally rely on daily dosing 7 days per week. The ACPHD defined treatment completion and success based on ingesting a set number of target doses. Any missed doses were added to the end of therapy, extending treatment duration.

### Routine treatment monitoring

Existing management protocols were in place during the evaluation period and developed by the local health department for routine programmatic purposes. The ACPHD protocol combined DOT 5 days per week (i.e., Monday to Friday), irrespective of in-person or vDOT, with weekend and holiday self-administration. Some patients submitted additional videos on weekends at their own discretion or at direction of staff in instances when weekday doses were missed; public health program staff ‘accepted’ or ‘rejected’ these additional video submissions according to local practice. Due to the focus on confirming observations only Monday to Friday, staff generally rejected any additional weekend video submissions unless there had been a missed weekday dose; consequently, local practice was focused on confirming adherence for 5 of 7 days of the week. Patients using vDOT were sent twice daily text message reminders in the absence of submitted videos and were prompted to document side effects prior to submissions. All patient data, servers, and transmissions were encrypted to protect patient privacy. At the time of study, the vDOT/DOT coordinator, and occasionally one other public health investigator, reviewed videos within 1 business day.

### Feasibility and effectiveness

We assessed the verified fraction, or proportion of total prescribed doses (inclusive of weekends, holiday, or other ‘self-administered’ doses) that were verified by observation (in-person or by video);^16^,^22^ time periods (e.g., weeks) were assessed as ‘in-person’ or ‘vDOT’ based on scheduled DOT modality. We also captured the proportion of prescribed doses that were self-administered during weeks monitored using vDOT vs. in-person DOT. We also measured maximum adherence, or the proportion of total prescribed doses taken under the assumption that all self-administered doses were ingested (along with verified observed doses). Differences in clinical and demographic characteristics comparing in-person DOT and vDOT were evaluated using two-sample *t*-tests and χ^2^ tests. We used multivariable logistic regression to assess the association of a priori selected relevant clinical and demographic factors associated with receipt of vDOT. All analyses were conducted in STATA v14 (Stata Corp, College Station, TX, USA).

## RESULTS

A total of 163 patients were treated for active TB during the study period, of which 100 (61%) were male, with a median age of 52 years (interquartile range [IQR] 34–67). More than 93% of patients were non-US-born, and the most commonly reported languages were English (53%), Chinese (Mandarin and Cantonese) (11%), Spanish (9%), and Vietnamese (9%). The majority of patients had pulmonary TB (*n* = 97, 60%), 42 patients (26%) had extrapulmo-nary TB, and the remainder had both pulmonary and extrapulmonary TB (*n* = 24, 15%); more than one third of patients had acid-fast bacilli (AFB) smear-positive disease at the onset of treatment (*n* = 59, 36%), and 22 patients (14%) had drug-resistant disease (17 had isoniazid monoresistance, while the rest had multidrug-resistant TB). Additional socioeconomic and demographic features are shown in Table 1.

**Table 1:** Patient characteristics

Baseline characteristics	All patients (*n* = 163) *n* (%)	Any vDOT (*n* = 94) *n* (%)	No vDOT^*^ (*n* = 69) *n* (%)	*P* value
Age, years, mean ± SD^”^	52.3 ± 19.7	46.1 ± 17.7	60.8 ± 19.3	<0.001^‡^
Female sex	63 (38.7)	41 (43.6)	22 (31.9)	0.129
Foreign-born	152 (93.3)	88 (93.6)	64 (92.8)	0.828
Ethnicity Hispanic	22 (13.5)	15 (16.0)	7 (10.1)	0.377
Race				0.148
Asian	121 (74.2)	65 (69.1)	56 (81.2)	
Native Hawaiian/Other Pacific Islander	6 (3.7)	5 (5.3)	1 (1.4)	
Black/African American	11 (6.7)	5 (5.3)	6 (8.7)	
White	19 (11.7)	15 (16.0)	4 (5.8)	
Unknown/not reported	6 (3.7)	4 (4.3)	2 (2.9)	
Limited or no English	75 (46)	39 (41.5)	36 (52.2)	0.149
Occupation				0.056
Health care worker	9 (5.5)	6 (6.4)	3 (4.3)	
Other worker	74 (45.4)	51 (54.3)	23 (33.3)	
Not seeking employment	62 (38.0)	29 (30.9)	33 (47.8)	
Unemployed	13 (8.0)	5 (5.3)	8 (11.6)	
Unknown	4 (2.5)	2 (2.1)	2 (2.9)	
Homelessness	4 (2.5)	2 (2.1)	2 (2.9)	0.753
Married/domestic partnership	102 (62.6)	59 (62.8)	43 (62.3)	0.954
Children in household	76 (46.6)	41 (43.6)	35 (50.7)	0.267
HIV-infected	5 (3.1)	4 (4.4)	1 (1.4)	0.019
Any drug use	5 (3.1)	3 (3.2)	2 (2.9)	0.688
Any alcohol use	2 (1.2)	2 (2.1)	0 (0.0)	0.099
Diabetes	49 (30.1)	24 (25.5)	25 (36.2)	0.759
TB drug resistance	22 (13.5)	12 (12.8)	10 (14.5)	0.926
AFB smear result	59 (36.2)	33 (35.1)	26 (37.7)	0.360
Any immunosuppression	12 (7.4)	8 (8.5)	4 (5.8)	0.512

* No vDOT represents a combination of patients with self-administered and in-person DOT.

† Age as of TB treatment start date.

‡ Statistically significant.

vDOT = video directly observed therapy; SD = standard deviation; AFB = acid-fast bacilli.

### Reach of vDOT

Approximately 58% (94/163) of participants received vDOT to monitor therapy for some portion of their TB treatment. All patients receiving DOT also had some portion of self-administration (e.g., weekend/holiday). Among those receiving vDOT, the median time on vDOT was 27.0 weeks (IQR 24.6–31.9). The mean number of prescribed doses was significantly greater during periods monitored by vDOT than those with in-person DOT (192 for vDOT vs. 149 for in-person DOT; *P* < 0.001). The proportion of patients receiving vDOT did not differ across the period of observation (49% quarter 1, 63% quarter 2, 50% quarter 3, 50% quarter 4; *P* = 0.737).

### Effectiveness of vDOT compared to in-person DOT

When considering the proportion of prescribed doses verified through observation, use of vDOT (or self-administration) led to an average 68.4% verified fraction vs. 53.9% using in-person DOT (or self-administration) (*P* < 0.001) (Table 2). A larger proportion of prescribed doses were considered ‘self-administered’ in the medical record during the time patients received in-person DOT (average 45.2%), compared to during the use of vDOT (average 23.8%; *P* < 0.001). By contrast, very few doses were documented as ‘missed’ during in-person DOT compared to periods using vDOT (average 1.3% vs. 3.4%, respectively; *P* < 0.001). Consequently, when considering self-administered doses as having been taken, the maximum adherence was considered to be higher when using in-person DOT than when using vDOT (98.6% vs. 90.3%, *P* < 0.001; Table 2).

**Table 2:** Primary outcomes by DOT strategy

Variable	No vDOT^*^ (*n* = 107) mean ± SD	Any vDOT (*n* = 94) mean ± SD	*P* value
Treatment adherence			
Adherence 1 (observed), %^†^	53.9 ± 25.6	68.4 ± 10.6	<0.001^§^
Median [IQR]	64.6 [57.8–68.4]	69.3 [66.4–71.2]	—
Adherence 2 (observed + self-administered),^‡^ %	98.7 ± 3.1	90.0 ± 9.9	<0.001^§^
Median [IQR]	100.0 [98.4–100.0]	93.5 [87.8–97.7]	—
Adherence 3 (observed + self-administered + rejected videos), %^¶^	—	95.9 ± 5.9	—
Median [IQR]	—	97.4 [94.6–99.6]	—
Number of prescribed doses	149.1 ± 101.2	192.0 ± 69.5	<0.001^§^
Median [IQR]	147 [61–198]	188 [171–223]	
Dose outcomes			
Proportion prescribed doses self-administered, %	45.2 ± 26.1	23.8 ± 11.5	<0.001^§^
Proportion prescribed doses ‘missed’, %	1.3 ± 3.1	3.4 ± 5.5	<0.001^§^
Video outcomes			
Number of rejected videos	—	5.7 ± 11.9	—
Median [IQR]	—	0.0 [0.0–5.0]	—
Video length, sec	—	43.5 ± 39.9	—
Median [IQR]	—	38.4 [5.4–55.2]	—
Video size, mb	—	9.3 ± 15.2	—
Median [IQR]	—	6.4 [4.1–8.8]	—
Time to video treatment start, weeks	—	6.9 ± 9.2	—
Median [IQR]	—	2.2 [1.1–10.0]	—

* Patients with no vDOT includes some patients who received exclusively self-administered therapy.

† Calculated as the observable fraction: doses that were observed/total number of doses.

‡ Calculated based on the assumption that patients take every dose that is not observed (including prescribed, dispensed, self-administered doses such as on weekends and holidays).

§ Statistically significant.

¶ Calculated based on crediting videos that were submitted by rejected videos (such as in cases where the observer could not visualize the pill).

DOT = directly observed therapy; vDOT = video DOT; SD = standard deviation; IQR = interquartile range.

We found similar successful treatment outcomes among those receiving any vDOT (96% completion/cure, 2% transferred to another program, and 2% death) and in-person DOT (90% completion/cure, 5% transferred to another program, and 4% deaths, *P* = 0.326), as well as similar microbiological outcomes (average 48 days’ time to culture conversion compared to 47 days among those receiving any vDOT and those receiving in-person DOT, respectively, *P* = 0.8429).

### Factors associated with vDOT usage

Individuals receiving some vDOT for treatment monitoring were younger than those that exclusively received in-person or self-administered therapy (average age, 46 years vs. 61 years; *P* < 0.001; Table 1). Adjusting for clinical and demographic factors, only age was significantly associated with vDOT use (adjusted odds ratio 0.05 comparing those >65 years to those <30 years, *P* < 0.001; Table 3). Among individuals receiving vDOT, the majority (61/93, 66%) began vDOT during the intensive phase. The median time to start vDOT was 2.2 weeks (IQR 1.1–10.0). Among those using vDOT, it represented the primary means of adherence verification; among these individuals, the median proportion of all prescribed treatment monitored using vDOT was 100% (IQR 79–100; mean 85%, standard deviation 23%).

**Table 3:** Multivariable logistic regression analysis for factors associated with receiving vDOT

Factors	aOR	(95% CI)
Age group, years		
<30 (reference)	1.00	Reference
30–50	0.31	(0.08–1.26)
51–65	0.27	(0.06–1.13)
>65	0.06	(0.01–0.27)
Sex		
Female (reference)	1.00	Reference
Male	0.78	(0.33–1.85)
Non-US-born		
No (reference)	1.00	Reference
Yes	1.58	(0.33–7.69)
English-speaking		
Non-English (reference)	1.00	Reference
English	1.03	(0.47–2.28)
Employment status		
Unemployed (reference)	1.00	Reference
Employed	2.25	(0.65–7.83)
Homelessness status		
No (reference)	1.00	Reference
Yes	0.34	(0.03–3.71)
Marital status		
Not married (reference)	1.00	Reference
Married	1.00	(0.43–2.35)
Children		
No (reference)	1.00	Reference
Yes	1.00	(0.98–1.01)
Drug use		
No (reference)	1.00	Reference
Yes	0.98	(0.95–1.02)
Alcohol use		
No (reference)	1.00	Reference
Yes	0.99	(0.96–1.02)
HIV status		
No (reference)	1.00	Reference
Yes	0.94	(0.77–1.15)
Diabetes status		
No/unknown diabetes (reference)	1.00	Reference
Has diabetes	0.79	(0.33–1.91)
TB drug resistance		
No (reference)	1.00	Reference
Yes	1.00	(0.99–1.01)
AFB smear result		
No (reference)	1.00	Reference
Yes	0.97	(0.95–1.0)

vDOT = video directly observed therapy; aOR = adjusted odds ratio; CI = confidence interval; AFB = acid-fast bacilli.

## DISCUSSION

In our prospective study of the implementation of vDOT under routine conditions in the United States, we found that a large, urban public health program (Alameda County, California) with a higher than average TB burden utilized vDOT for treatment monitoring in more than half of patients. These findings suggest shifting paradigms for adherence monitoring in TB control programs that traditionally relied more heavily on in-person DOT. Moreover, significantly more prescribed doses of TB treatment were verified utilizing vDOT vs. in-person DOT (68% vs. 54%; *P* < 0.001). Our study is unique in assessing programmatic vDOT implementation, rather than under controlled study conditions. Our findings were similar to those identified under study settings, where vDOT achieved greater medication verification than in-person DOT.^16^,^22^,^27^–^29^

Among the goals of DOT is to verify prescribed TB treatment. However, our results indicated that under routine circumstances, only slightly more than half of all TB treatment doses are verified using in-person DOT. This finding reflects the logistical realities of restricting in-person DOT services to business days and is a growing concern as TB programs globally shift to daily (7 days/week) of therapy.^15^,^30^–^32^ Paradoxically, daily in-person DOT can have a negative impact on adherence for some and its implementation can be challenging, particularly in resource-constrained settings.^33^,^34^ While a larger proportion of prescribed doses were verified using vDOT, the potential for vDOT has not been fully realized. In Alameda, patients were given instructions to self-administer weekend doses, largely for programmatic reasons, including to be consistent with typical ‘dose counting’ for in-person DOT during days where self-administration (on weekends) was the norm. Furthermore, during the study period, a single healthcare worker was responsible for watching the majority of vDOT videos, which influenced local protocols for vDOT usage on weekends.

In 2016, CDC/ATS/IDSA guidelines were updated to indicate a preference for daily treatment regimens over intermittent dosing regimens (i.e., twice per week or three times per week dosing) based on evidence for greater regimen effectiveness;^15^ globally, WHO recommendations have similarly prioritized daily therapy.^35^ This recommendation change has practical implications for public health TB programs regarding strategies for monitoring adherence, particularly those utilizing in-person DOT. Consequently, US guidelines included the option for dosing 5 days per week using DOT (e.g., 40 intensive phase doses and 90 continuation phase doses), while also acknowledging that “there are no studies that compare 5 with 7 daily doses.”^15^ Our real-world data, however, highlights implementation challenges related to in-person DOT. In Alameda, medications were typically prescribed 7 days per week, with adherence monitoring restricted to only a subset of days. We found that 45% of treatment doses were self-administered when utilizing in-person DOT. Prior data suggest that adherence reports with self-administration can be over-estimated.^36^,^37^ We found that maximum adherence could have been as high as 98.6% under the assumption that all self-administered doses were taken, but adherence estimates were as low as 53.8% when considering only verified ingestion by in-person DOT.

On the other hand, while there were fewer self-administered doses with vDOT, we found that less than three quarters of prescribed doses were confirmed by observation, attributable to implementation protocols. Recognizing the improved effectiveness of daily therapy in the treatment of active TB, our results suggest that additional guidance on assessing treatment adherence and completion may be warranted. vDOT offers the opportunity to verify treatment 7 days per week; future research is needed to assess whether this closer monitoring could improve clinical outcomes.^38^

Our study had several important limitations. This study was limited to vDOT evaluation at one health department; vDOT usage may differ across areas or settings. Nonetheless, ACPHD is representative of many large, urban public health TB programs, particularly in the United States. This study was conducted in a program with prior pilot program vDOT experience; thus, we did not identify significant changes in vDOT usage across the first and last quarters of the study period. The reach and feasibility of vDOT implementation may differ for programs introducing vDOT for the first time. Finally, our study concluded before the onset of the COVID-19 pandemic. Whether vDOT uptake changed in the setting of public health restrictions and physical distancing mandates should be investigated.

Our study is among the first to provide insights on key aspects of the real-world implementation of vDOT under routine (i.e., non-study) conditions for a program using daily TB therapy. We found that vDOT usage was high, with 58% (94/163) of patients utilizing vDOT at some point during treatment. Our data provide practical evidence to support programmatic paradigm shifts away from exclusive reliance on in-person DOT; 57% of vDOT recipients (54/94) received vDOT exclusively without any in-person DOT monitoring. Our results further highlight that vDOT implementation can be achieved early in patients’ treatment, in contrast to prior studies in which verification of good adherence was required prior to vDOT initiation. The majority of patients in Alameda initiated vDOT during the intensive phase, with a median time to vDOT start of less than 2.5 weeks. While the CDC toolkit and California guidelines on implementation considerations for vDOT suggest that patients successfully complete initial weeks of in-person DOT, our data suggest vDOT can be used exclusively in selected patients and with a high degree of adherence.^7^,^25^,^26^ This study provides important insights into selecting patients who routinely receive vDOT. We found that vDOT implementation by the ACPHD was more common among younger individuals. Our findings may reflect a greater comfort among younger individuals with technology usage, or a perception among healthcare workers that younger individuals are better suited for vDOT. Nonetheless, 10% of individuals over 65 years old also utilized vDOT, suggesting that this modality is also feasible among the elderly. Future studies should aim to understand how to increase vDOT reach in older populations. Alternatively, implementing vDOT in some patients, even those who are younger, can allow public health programs to focus in-person TB efforts on patients that require closer supervision or direct nursing care.

Overall, our study contributes to the growing literature on the programmatic implementation of vDOT as an alternative to in-person DOT for TB treatment. We expand on prior efforts by reporting data from among the largest prospectively collected cohorts of routine, programmatic implementation of vDOT. Prior studies have suggested that vDOT may be a feasible, effective, and cost-effective approach to verification of treatment under study conditions.^16^,^19^,^23^,^33^,^39^ Given the relatively recent adoption of vDOT within public health TB programs, the CDC and California State guidelines acknowledge the need for monitoring and evaluation to provide data on vDOT reach and effectiveness.^7^,^25^,^26^ Our findings identified a high rate of real-world vDOT reach, adoption and effectiveness in a busy, urban setting, as well as the need for continued programmatic monitoring over time and across other settings to inform TB treatment guidelines, policy, and resource allocation in TB programs.
